# Calcineurin Orchestrates Hyphal Growth, Septation, Drug Resistance and Pathogenesis of *Aspergillus fumigatus*: Where Do We Go from Here?

**DOI:** 10.3390/pathogens4040883

**Published:** 2015-12-16

**Authors:** Praveen R Juvvadi, William J Steinbach

**Affiliations:** 1 Department of Pediatrics, Division of Pediatric Infectious Diseases, Duke University Medical Center, Durham, NC 27710, USA; E-Mail: bill.steinbach@duke.edu; 2 Department of Molecular Genetics and Microbiology, Duke University Medical Center, Durham, NC 27710, USA

**Keywords:** *Aspergillus fumigatus*, calcineurin, hyphal growth, septation, virulence, FK506, cyclosporine A, phosphorylation, drug target, drug resistance

## Abstract

Studies on fungal pathogens belonging to the ascomycota phylum are critical given the ubiquity and frequency with which these fungi cause infections in humans. Among these species, *Aspergillus fumigatus* causes invasive aspergillosis, a leading cause of death in immunocompromised patients. Fundamental to *A. fumigatus* pathogenesis is hyphal growth. However, the precise mechanisms underlying hyphal growth and virulence are poorly understood. Over the past 10 years, our research towards the identification of molecular targets responsible for hyphal growth, drug resistance and virulence led to the elucidation of calcineurin as a key signaling molecule governing these processes. In this review, we summarize our salient findings on the significance of calcineurin for hyphal growth and septation in *A. fumigatus* and propose future perspectives on exploiting this pathway for designing new fungal-specific therapeutics.

## 1. Introduction

Invasive fungal infections are one of the most common causes of death in immunocompromised patients [1]. Calcineurin is a target of immunosuppressive agents, tacrolimus (FK506) and cyclosporine A (CsA), mediated via their respective immunophilins, FKBP12 and cyclophilin A [2,3]. Genetic studies in fungi have established the molecular mechanism of calcineurin inhibition by the CsA-cyclophilin A and FK506-FKBP12 complexes [2,4,5]. However, these currently available anti-calcineurin agents are not ideal for treating invasive fungal infections due to their host calcineurin cross-reactivity and subsequent immunosuppressive effects [3]. Therefore, an in depth understanding of calcineurin and its functions in fungi is essential to exploit the calcineurin pathway to formulate novel fungal-specific targeting approaches.

Calcineurin is a heterodimer comprised of a catalytic (CnaA) and regulatory (CnaB) subunit [6]. It is also known as protein phosphatase 2B and is activated through the binding of Ca^2+^-calmodulin (CaM) [7]. Among the known serine/threonine protein phosphatases, calcineurin is the only phosphatase that requires Ca^2+^ and CaM for its enzymatic activity and exhibits restricted substrate specificity [7,8,9]. Though highly conserved from the lower eukaryotes to humans, calcineurin performs diverse functions in different organisms [10]. For instance, in the yeasts *Saccharomyces cerevisiae* and *Schizosaccharomyces pombe*, it regulates adaptation to a variety of environmental stresses, cation homeostasis, morphogenesis, cell wall integrity, and mating [11,12,13,14,15,16,17,18,19]. In the pathogenic yeasts *Cryptococcus neoformans* and *Candida albicans,* calcineurin regulates growth at alkaline pH, elevated temperature, membrane stress, dimorphism, mating and virulence [20,21,22,23,24]. In filamentous fungi, it regulates hyphal growth, stress adaptation, sclerotia and appresoria development and cell wall integrity [25,26,27,28,29,30,31,32,33,34].

As a drug target, calcineurin’s roles have been extensively investigated in human pathogens such as *C. neoformans*, *C. albicans* and other *Candida* species, *A. fumigatus*, and *Mucor circinelliodes* [35,36,37,38,39]. Understanding the calcineurin signaling network in the various fungal pathogens is beneficial for designing new targeted therapeutics. Filamentous fungi have a single set of genes encoding the calcineurin catalytic and regulatory subunits and are therefore ideal model systems to study the role of these subunits *in vivo*. Critically investigating the calcineurin pathway in *A. fumigatus* to identify residues indispensable for calcineurin activity *in vivo* will pave the way for devising new drug targets for combating invasive aspergillosis. In this review, we summarize our latest findings on calcineurin functions in this human pathogen and how these relate to its cellular localization and influence on the various downstream events and targets.

## 2. Calcineurin Is Essential for Hyphal Extension and Septation

**Figure 1:**
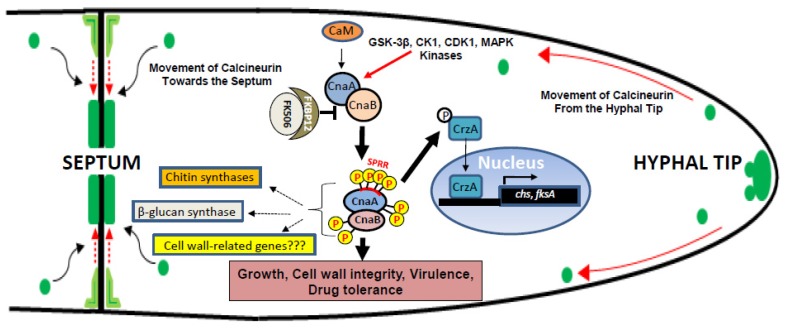
Schematic model of calcineurin localization and function in *A. fumigatus*. Calcineurin localizes to puncta which are motile and move retrograde from the hyphal tip to the point of septation and localize on either sides of the septum as a disc around the septal pore. Comprised of the catalytic (CnaA) and the regulatory subunit (CnaB), calcineurin binds to calmodulin (CaM) and is activated through phosphorylation by kinases (GSK-3β, CK1, CDK1, MAPK). Phosphorylation occurs at the Serine Proline Rich Region (SPRR) and the C-terminus. CnaB also undergoes phosphorylation at the N-terminus. Calcineurin is inhibited by the binding of the immunosuppressant-immunophilin complex (FK506-FKBP12). Phosphorylated calcineurin complex may dephosphorylate the transcription factor CrzA and translocate it into the nucleus to activate the transcription of cell wall biosynthesis related genes (*chs* genes and *fksA*). Similarly, the phosphorylated calcineurin complex may also interact with cell wall proteins directly in a phosphorylation-dependent manner to regulate cell wall integrity, virulence and drug resistance.

Previous studies from our laboratory and from others have shown that deletion of the catalytic subunit of *A. fumigatus* calcineurin (*cnaA*) resulted in blunted, hyperbranched and irregularly septated hyphae, indicating the requirement of calcineurin for proper hyphal extension and regular septation [40,41,42]. Double deletion of both the subunits (*cnaA* and *cnaB*) caused more severe defects in germination and septation [42]. To better understand how and to what extent calcineurin controls septum formation and hyphal growth, more detailed studies on visualizing the localization patterns of CnaA and CnaB in hyphae demonstrated their localization at the active points of hyphal growth—the hyphal tip and hyphal septum [42,43]. Calcineurin localization was also observed in small punctate structures that were motile and moved retrograde from the hyphal tip to the point of septation (Figure 1) [42]. To address calcineurin’s role during septum formation, we visualized the actin contractile ring during septum formation by co-localization of fluorescently labeled CnaA and actin (LifeAct) [42]. Localization studies with actin also confirmed its co-localization with calcineurin early during the septation process and after the formation of mature septa. This result indicates that the calcineurin complex participates in early septation process and may be used for activation of the cell wall biosynthetic components. No variations were noted in the contractile actin ring formation in the Δ*cnaA* Δ*cnaB* double mutant strain, indicating that the septation defects may be caused during the primary or secondary wall formation. The cell wall defects observed in the calcineurin deletion strains could largely be due to inactivation of the PKC (Protein kinase C) pathway and the Crz1 transcription factor, which is activated by calcineurin.

Interestingly, the calcineurin deletion strains do form septa, but very irregularly and with shorter apical, subapical and basal hyphal compartments. While the septa in the Δ*cnaA* and Δ*cnaB* strains were similar, the double mutant showed a large percentage of incomplete septa or wavy septa [42]. Previous reports on the characterization of the chitin synthase double mutant (Δ*chsA* Δ*chsC*) and a Δ*chsB* single mutant in *Aspergillus nidulans* also showed irregular septation and wavy/incomplete septa [44,45] resembling the calcineurin double mutant. Recent deletion analysis of multiple genes belonging to class I (*chsA*, *chsB*, *chsC* and *chsG*) and class II (*chsF*, *chsD*, *csmA* and *csmB*) chitin synthase family in *A. fumigatus* also revealed defects in hyphal morphology, conidiation and cell wall organization [46]. Although the specific role of calcineurin during septum formation is yet unknown, similar septation and cell wall organization defects observed with the chitin synthase deletion strains in *A. nidulans* and *A. fumigatus* implicates calcineurin as playing a role in the activation of chitin synthases or chitin and β-glucan assembly at the hyphal septum (Figure 1). Supporting this notion, we also found that the calcineurin double mutant has less β-glucan at the septa during early periods of growth when compared to the single mutants [42].

## 3. Septal Localization: A Key to Calcineurin Function

Hyphae of *A. fumigatus* extend and invade host lung tissue in order to cause invasive aspergillosis. Septation is an important event for hyphal extension and the calcineurin complex appears to regulate this process through a yet undefined mechanism in filamentous fungi. Although the catalytic subunit (CnaA) localizes at the hyphal septum, it alone is unable to complement for hyphal growth and requires the regulatory subunit (CnaB) for the activity and function at the hyphal septum [42]. Catalytic null mutations generated in CnaA do not complement the hyphal growth defect but do not affect the localization of the complex at the hyphal septum [42,47]. Though the complex localizes at the hyphal septum, it needs to be enzymatically active to regulate proper hyphal growth. Taken together, both localization and activity of the calcineurin complex at the hyphal septum are important for its function (Figure 1).

To understand the mechanistic basis for calcineurin localization and identify critical domains essential for its localization and function at the hyphal septum, we performed site-directed mutagenesis of conserved residues in the linker between the CnaA catalytic domain and CnaB binding helix (T359P, H361L, L365S), the PxIxIT binding motif (352NIR354-AAA), CnaB binding helix (V371D), calmodulin binding domain (CaMBD; 442RVF444-AAA) and the unique serine-proline rich region (SPRR; 406-SPSAPSPPLP-417) in the linker region between the CnaB binding helix and CaMBD of CnaA [42,47]. These mutations revealed that only the PxIxIT substrate binding motif residues 352NIR354 are required for septal localization, confirming that calcineurin localizes at the septum through interaction with as yet unknown substrate(s) at the septum.

To examine if the immunosuppressant and immunophilin complex is also required for localizing the CnaA-CnaB complex at the hyphal septum, we also treated it with FK506 and CsA and examined it for any change in the calcineurin localization pattern. FK506 or CsA treatment did not alter the localization of the calcineurin complex from the septa, indicating that localization of the complex at the septa is not dependent on binding to the immunophilins [42]. However, since FK506 inhibits the activity of calcineurin, it became evident that functional activity of the calcineurin complex is absolutely required at the hyphal septum in order to regulate proper hyphal growth. In support of this observation, we also recently showed that FKBP12 localized to the hyphal septum only in the presence of FK506 [48].

Since the calcineurin complex selectively concentrates at the hyphal septum, we are pursuing future work to identify the targets of calcineurin at the hyphal septum to clarify the exact function of this complex in regulating septation and hyphal growth. We expect these studies will lead to identification of other targets of calcineurin.

## 4. Calcineurin Phosphorylation: A Unique Modification

Various functional aspects of calcineurin have been studied in several model organisms and its functional domains described, but phosphorylation as a mechanism of calcineurin function was never before examined in a human pathogen. Our phosphoproteomic analyses revealed *A. fumigatus* CnaA phosphorylation *in vivo* at a unique cluster of serine and proline residues, that we termed the Serine Proline Rich Region (SPRR), that is specific to filamentous ascomycetes (Figure 1) [47]. Phosphorylation at the four serine residues in proximity to the proline residues may induce secondary structure conformation in the molecule, facilitating interactions with other proteins. Furthermore, mutation of the phosphorylated serine residues in the SPRR to block the phosphorylation of CnaA *in* *vivo* caused increased branching and reduction in hyphal growth [47], confirming the significance of phosphorylation for calcineurin function and activity in regulating hyphal growth.

Interestingly, phosphorylation was reduced in both the catalytic and the regulatory calcineurin subunits following treatment with FK506 [47]. It is possible that the FK506-FKBP12 complex may indirectly cause an inhibitory effect on the kinase that phosphorylates calcineurin at the SPRR and also at the N-terminus of CnaB. Based on the phosphorylation of CnaA residues in both the SPRR and the C-terminus, we speculated that more than one kinase is responsible. Proline-directed kinases such as GSK-3, CDK1 and MAP kinase were able to phosphorylate all four serine residues in the SPRR (Figure 1) [47]. While GSK-3β alone phosphorylated Ser413 in the SPRR, CK1 alone phosphorylated Ser406, and a combination with GSK-3β led to phosphorylation of other serine residues (Ser408 and Ser410), revealing that the prephosphorylation of the Ser406 residue by CK1 may trigger the subsequent phosphorylation of Ser408 and Ser410. Inhibitors for GSK-3β and CK1 blocked phosphorylation at Ser406 only, but not the other serine residues, leading to the notion that other yet unknown kinase may also phosphorylate the CnaA SPRR *in vivo*. The observed phosphorylation of CnaA SPRR by CDK1 and MAP kinase *in* *vitro* strengthens this possibility. However, further analyses are required to specifically identify the kinase/s involved in CnaA phosphorylation. In another recent study, we also showed differential phosphorylation of calcineurin following treatment with two echinocandin antifungals, caspofungin and micafungin [49]. We found that calcineurin phosphorylation at the SPRR is important for regulation of “paradoxical growth” at higher concentrations of caspofungin. This suggests that phosphorylation of calcineurin could be the mechanism underlying the phenotypic paradoxical growth. Further work is required to clearly understand how these dynamic changes in the phosphorylation status of CnaA following treatment with the two echinocandins influences calcineurin’s inactivation/activation and its subsequent interaction with downstream targets.

## 5. Calcineurin as an Antifungal Drug Target: Future Perspectives

Due to limitations in the currently available antifungal armamentarium, novel therapeutic strategies are needed to combat invasive aspergillosis [50,51]. Calcineurin inhibitors are attractive as new antifungal agents due to their specific mode of action from other antifungal classes (polyenes, triazoles, echinocandins) that would target the top of a critical cell signaling pathway, efficacy against emerging azole- and echinocandin-resistant strains [52], and synergistic nature with existing antifungals such as the echinocandin antifungal caspofungin [53]. Moreover, the observed “paradoxical growth effect” at higher concentrations of caspofungin is abolished in the presence of FK506 and by genetic mutations of calcineurin, implicating interaction of calcineurin with cell wall biosynthetic machinery [54]. However, currently available calcineurin inhibitors bind to immunophilins, leading to host immune suppression and compelling the need for designing non-immunosuppressive calcineurin inhibitors. In addition, due to the highly conserved nature of calcineurin molecule, it is important to obtain crystal structure of *A. fumigatus* calcineurin in order to design strategies to specifically target calcineurin in the fungal pathogen without affecting the host. Identification of the unique SPRR in filamentous fungal calcineurin is one such domain that can be pursued for potential targeting. Our finding of differential phosphorylation of calcineurin at the SPRR in the presence of micafungin and caspofungin indicates the probability of these antifungals inhibiting the phosphorylation of calcineurin by blocking the kinase responsible for phosphorylation. Identification of the kinase and inhibiting its activity can be another potential alternative. Additional strategies should involve characterization of the whole calcineurin interactome and phosphoproteome to define specific cell wall biosynthesis related interactors that are regulated by calcineurin through dephosphorylation. Data from such analyses may lead to the identification of potential new alternative targets and the design of better dual targeting strategies. For instance, our recent characterization of the two major heat shock proteins in *A. fumigatus* (Hsp90 and Hsp70) revealed their interaction with calcineurin [55] and a positive interaction between FK506 and geldanamycin (Hsp90 inhibitor), leading to fungicidal effect was noted [52,56,57].

## 6. Conclusions

As a critical phosphatase, calcineurin may regulate several downstream targets through dephosphorylation and contribute to the activation/deactivation of numerous effector proteins and possibly other signaling pathways. Calcineurin control over hyphal morphogenesis, septation, cell wall integrity, and stress adaptation, may all contribute to fungal virulence. Despite being a drug target, the immunosuppressive nature of the current calcineurin inhibitors is an obstacle to overcome, and designing non-immunosuppressive anti-calcineurin agents that are fungal-specific is challenging. Over the last decade, our studies have provided new insights into its role in hyphal growth and septation. Exploiting some of the unique aspects of fungal calcineurin, such as phosphorylation or identification of its interactions with fungal specific structures such as the cell wall and septum machinery regulators, may lead us to new targets to explore.
